# Maternal Immune Activation and the Development of Dopaminergic Neurotransmission of the Offspring: Relevance for Schizophrenia and Other Psychoses

**DOI:** 10.3389/fpsyt.2020.00852

**Published:** 2020-08-21

**Authors:** Argel Aguilar-Valles, Brandon Rodrigue, Edna Matta-Camacho

**Affiliations:** Department of Neuroscience, Carleton University, Ottawa, ON, Canada

**Keywords:** maternal infection, schizophrenia, dopamine, animal models, cytokines, IL-6, iron, leptin

## Abstract

Prenatal infections have been linked to the development of schizophrenia (SCZ) and other neurodevelopmental disorders in the offspring, and work in animal models indicates that this is to occur through the maternal inflammatory response triggered by infection. Several studies in animal models demonstrated that acute inflammatory episodes are sufficient to trigger brain alterations in the adult offspring, especially in the mesolimbic dopamine (DA) system, involved in the pathophysiology of SCZ and other disorders involving psychosis. In the current review, we synthesize the literature on the clinical studies implicating prenatal infectious events in the development of SCZ. Then, we summarize evidence from animal models of maternal immune activation (MIA) and the behavioral and molecular alterations relevant for the function of the DAergic system. Furthermore, we discuss the evidence supporting the involvement of maternal cytokines, such as interleukin 6 (IL-6) and leptin (a hormone with effects on inflammation) in mediating the effects of MIA on the fetal brain, leading to the long-lasting effects on the offspring. In particular, IL-6 has been involved in mediating the effects of MIA animal models in the offspring through actions on the placenta, induction of IL-17a, or triggering the decrease in non-heme iron (hypoferremia). Maternal infection is very likely interacting with additional genetic and environmental risk factors in the development of SCZ; systematically investigating how these interactions produce specific phenotypes is the next step in understanding the etiology of complex psychiatric disorders.

## Introduction

We are currently undergoing a SARS-CoV-2 pandemic, which like previous viral outbreaks [e.g., Zika (1)] can leave behind sequelae of health complications, including direct effects in the nervous system (2) and alterations of brain development if infections occur during perinatal stages.

Indeed, maternal infection has been identified as a risk factor for several neurodevelopmental disorders such as cerebral palsy, intellectual disability, autism spectrum disorder (ASD), bipolar disorder (BD), and schizophrenia (SCZ) (3–9). We will focus on reviewing the effects of maternal infection on the dopaminergic neurotransmitter system and the link with psychosis, particularly SCZ.

SCZ is one of the top leading causes of disability worldwide (10) and the seventh most costly medical illness in modern society (11, 12). SCZ is characterized by psychotic symptoms such as delusions and hallucinations (also known as the positive symptom dimension); alterations in drive and volition, including lack of motivation, blunted affect, social withdrawal, and reduction in spontaneous speech (the negative symptom dimension) and alterations in neurocognition, including difficulties in memory, attention, and executive functioning (the cognitive symptom dimension) (13–15).

The positive symptoms of SCZ overlap with different psychiatric disorders. Indeed, psychosis is also frequent during mood episodes in BD, severe depression, substance use disorder and neurodegenerative disorders (16–18). Intriguingly, some SCZ-like psychopathological abnormalities (i.e., paranoid delusional thinking and auditory hallucinations) are expressed in an attenuated form in 5–8% of the otherwise healthy population, especially in individuals with schizotypal or schizoid personality traits (13, 19). This extensive overlapping of symptoms and genetic risk factors with other psychiatric and neurological conditions is suggestive of a common underlying neuropathophysiology for these disorders, which, rather than discrete diagnoses, may represent a continuum that extends to the general population (13, 19, 20).

## The Dopamine Theory Of SCZ And Psychosis

**Figure 1:**
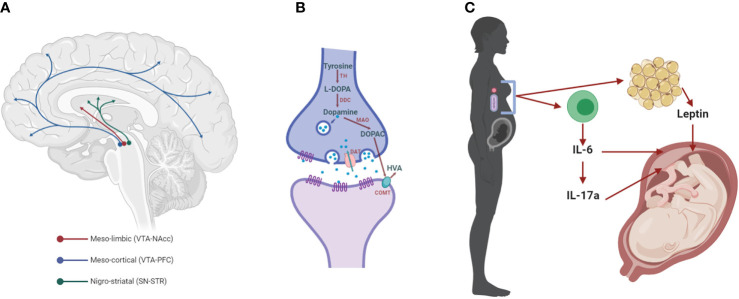
The dopaminergic system and mediators of maternal immune activation. **(A)** The meso-limbic DA neurons have their cell bodies in the ventral tegmental area (VTA) and terminals innervate the nucleus accumbens (NAcc). Other VTA neurons project to the prefrontal cortex (PFC), constituting the meso-cortical system. The nigro-striatal DA neurons lie in the substantia nigra (SN) and project to the dorsal striatum (STR). **(B)** Dopaminergic synapse, where dopamine is synthesized by the conversion of tyrosine into L-3,4-dihydroxyphenylalanine (L-DOPA) by the enzyme tyrosine hydroxylase (TH). L-DOPA is then converted to dopamine by the L-DOPA decarboxylase (DDC). Once packaged in synaptic vesicles and released to the extracellular space, dopamine can act on its receptors (DRs) on the post- and pre-synaptic membrane. Dopamine neurotransmission is terminated when the dopamine transporter (DAT) reuptakes the neurotransmitter to the pre-synaptic side, where it can be metabolized into 3,4-dihydroxyphenylacetic acid (DOPAC) by the monoamine oxidase (MAO) and homovanillic acid (HVA) by the catechol-O-methyl transferase (COMT). **(C)** Maternal immune activation (MIA) with bacteria or viruses leads to the activation of immune cells that release cytokines, including interleukin 6 (IL-6) and, in turn, IL-17a. Both of these cytokines affect brain development in the fetus, increasing the risk for neurodevelopmental disorders, such as SCZ (SCZ). These cytokines can act indirectly on the placenta, or in the case of IL-6 through the induction of hypoferremia, a reduction in circulating non-heme iron. Adipose tissue can also release hormones such as leptin, which affects fetal development.

The classical dopamine (DA) hypothesis of SCZ (21) states that the hyperactivity of the DA system is responsible for the symptoms of the disorder. More recently, this hypothesis was elaborated to include the proposal that the hyperactivity of the mesolimbic DA system (**Figure 1A**) contributes to positive symptoms in SCZ. Meanwhile, impaired function of the DA system in the prefrontal cortex (PFC, **Figure 1A**) contributes to the cognitive symptom dimension (22, 23).

The DA hypothesis of SCZ derives, in part, from the identification of the mechanisms of action of antipsychotics, many of which act as DA receptor 2 (D2 receptor) blockers (15). Furthermore, pharmacological studies show that a single exposure to amphetamine (AMPH), a stimulant drug that increases extracellular levels of DA in striatal and cortical regions *via* release and reverse transport (24, 25), evokes or exacerbates positive symptoms in SCZ patients at doses which do not induce psychosis in healthy subjects (26–28). Imaging studies demonstrate that a significant number of non-medicated SCZ patients show marked elevation of AMPH-induced striatal dopamine release in comparison to healthy volunteers (29–31). This response correlates significantly with the emergence or worsening of positive symptoms (31–35).

Understanding the etiology of SCZ is an active area of research. However, evidence accumulated in the last three decades on environmental risk factors that affect early neurodevelopment during pregnancy has led to the proposal of the neurodevelopmental hypothesis for SCZ (36–38). In this sense, accumulating evidence suggests that perinatal insults also contribute to an increase risk of developing BD (9), particularly those cases with psychoses (8).

## Neurodevelopmental Etiology Of SCZ And Other Psychiatric Disorders

SCZ has been hypothesized to have a neurodevelopmental origin (22): an outcome of an aberration in developmental processes within the brain, which begins long before the onset of the clinical symptoms (36, 39). There are numerous independent lines of evidence supporting this hypothesis. For example, there is a conspicuous absence of gross physical damage or signs of progressive neurodegeneration in SCZ (22, 39). Besides, children that go on to develop SCZ present behavioral, physical and brain morphological alterations, before the clinical onset of psychosis (15, 36, 39–41).

Finally, individuals who develop SCZ are more likely to have experienced pre- or perinatal adverse events (22, 42), or adolescent disturbances in brain development, compared to control individuals (36, 39, 41, 43). These adverse events include intrauterine growth retardation, pregnancy and birth complications (44), nutritional deficiencies (45, 46) maternal stress (47), and maternal infections (48).

There is also mounting evidence for the role of neurodevelopmental disturbances in the etiology of BD, as thoroughly reviewed by (9). In this regard, there is high co-morbidity between BD and other developmental disorders such as attention-deficit/hyperactivity disorder (ADHD) and ASD (9). Remarkably, there are increased rates of BD due to obstetric complications, cesarean section birth and perinatal infection (8, 9, 49, 50).

## Maternal Infection, SCZ, and Other Neurodevelopmental Disorders

Ecological studies, including those based on the subjective report of illness, suggest that SCZ is more prevalent in the offspring of women that were pregnant during periods of influenza epidemics (51, 52), as well as other types of infections, including diphtheria, pneumonia, measles, varicella zoster, mumps and poliovirus (4, 53–56). Similarly, SCZ is more prevalent among individuals born to pregnancies that occur during winter, a season associated with an increased frequency of respiratory infections (36, 51, 57). The main limitation of these studies is that “exposure to infection” was defined solely by the fact that the individual was pregnant during the time of the epidemic (i.e., based on the date of birth of the offspring).

It was later shown that SCZ in the offspring is significantly associated with maternal infections using individual biomarkers of illness in the maternal serum or clinical diagnoses (4). These included respiratory infections (58), influenza (59, 60), rubella (61, 62), *Toxoplasma gondii* (63, 64), herpes simplex virus-2 (HSV-2) (65, 66), maternal genital or reproductive infections (67), and maternal bacterial infections (68). Some of these studies used a broad definition of psychosis, where both non-affective (e.g., SCZ) and affective (e.g., major depression or BDs with psychotic features) psychiatric disorders were included (62, 66, 69). This suggests that maternal infection may be involved in the development of psychotic features that may not be necessarily restricted to those that characterize SCZ, but several other disorders as well. Indeed, MIA involving influenza has been linked to BD (9) [and Toxoplasma gondii infections to a lesser extent (70)], especially for those patients that also develop psychotic features (8).

What remains unclear from these studies is the critical stage(s) of gestation during which the developing brain may be more vulnerable to this prenatal insult. Indeed, those studies that have tried to dissect a specific trimester of gestation where vulnerability to MIA may be increased, have provided evidence for all three trimesters (58, 59, 68, 69, 71, 72). Overall, effect sizes of prenatal infection across gestation and development of SCZ in the offspring range from 1.5 to 7 for different infections (73), suggesting the existence of additional factors that confer vulnerability or resilience (6).

The wide variety of infections associated with SCZ and BD with psychosis suggests that there may be a common factor underlying increased susceptibility (74). Therefore, it has been hypothesized that maternal immune activation (MIA), and the inflammatory mediators released following all types of infections (4), may be fundamentally involved. Epidemiological studies have provided some evidence supporting this hypothesis. Increased levels of maternal pro-inflammatory cytokines, specifically interleukin (IL)-8 (72), tumor necrosis factor (TNF)α (69, 71, 75, 76), IL-6 (71, 75, 76), C-reactive protein (77) are associated with a higher risk of psychosis or SCZ in the offspring. Several animal models have been developed to investigate the immunological and neurobiological link between MIA and altered behavior in the offspring, with heavy emphasis in behavioral alterations.

## Animal Models of Maternal Infection

Initial approaches used prenatal infection with an influenza virus [at gestational day (GD) 9 in mice], followed by the application of a battery of behavioral tests relevant to SCZ in the adult offspring (78). These studies showed that the adult offspring of infected mothers presented, compared to the offspring of control dams, decreased social interaction, reduced exploration in the open field, impaired performance in the novel object test, indicative of impaired working memory as observed in SCZ and diminished PPI of acoustic startle (78). These behavioral alterations are analogous to aspects of SCZ.

### Viral Mimetic Poly I:C

Further studies investigated the consequences of MIA using molecular immunogens in rats and mice. The viral mimic polyinosinic:polycytidylic acid (poly I:C) has been used to stimulate the maternal immune system (with one or multiple injections), at several stages of pregnancy in mice or rats, ranging from GD 8.5 until GD 18.5. The effects of these prenatal treatments have been extensively reviewed elsewhere (6, 7, 79–81); thus, we will focus on those consequences more closely relevant for psychosis. Prenatal stimulation with poly I:C induced deficits in an operational measure of sensorimotor gating (82), pre-pulse inhibition of acoustic startle and increased sensitivity to the locomotor activating effects of cocaine, AMPH and methamphetamine, whose locomotor effects depend on the mesolimbic DA system (83–105).

An overall trend regarding these two phenotypes is one where PPI deficits are more consistently observed when MIA occurs at gestational stages earlier than GD 16 in both mice and rats. At the same time, hyper-responsiveness to activators of the mesolimbic dopaminergic system appears when challenging the mothers at any developmental age [reviewed in (79)].

### Models of Bacterial Infections

The role of bacterial infection has also been investigated by using the Gram-negative bacterial cell wall component, lipopolysaccharide (LPS). In rats, injections at several stages of gestation, ranging from GD 9 until birth, induced, in the offspring, impairments in PPI, and increases in sensitivity to the locomotor effects of AMPH (106–117). LPS has also been administered either in alternate days (118) or daily throughout pregnancy (119, 120). Similarly to acute LPS administration, these chronic prenatal treatments also induced impairments in PPI (118–120).

### Other Models of Inflammation

Turpentine (TURP) is an inflammatory agent whose injection [intramuscular (i.m.)] produces localized necrotic damage (121) and the sequential induction of TNFα and IL-1β at the site of injury, which trigger IL-6 release into the circulation (122, 123). Using TURP at GD 15 or 18 in rats, we found that an earlier challenge with TURP induces greater maternal inflammatory response compared to later in gestation (124). Furthermore, this difference in the inflammatory response during pregnancy correlates with the effect on the offspring, such that treatment at GD 15 induces impairment in PPI and hyper-responsiveness to AMPH, while the same treatment at GD 18 does not affect any of these behaviors (124).

Overall, some of the alterations in behavior induced by either polyI:C or LPS, have been shown to appear in the adult but not in the juvenile offspring (84, 93, 120, 125), as occurs in SCZ patients. Also supporting the validity of the models toward the disorder is the observation that a number of these alterations, including deficits in PPI, were shown to be reversed by either acute or chronic treatment with several antipsychotic drugs in adult or adolescent animals [i.e., haloperidol, chlorpromazine, olanzapine, risperidone or clozapine, which constitute the primary pharmacological treatment for psychotic illness (78, 83–85, 89, 119, 126–132)].

## Effects of Maternal Infection on Dopamine Neurotransmission in Mouse Models

Given the central role of DA neurotransmission in SCZ, the findings on the exaggerated locomotor response to AMPH and other drugs that stimulate DAergic neurotransmission following MIA, and the effectiveness of antipsychotic treatments to reverse MIA effects, several studies investigated the effects of prenatal immune activation on this neurotransmitter system. One often used approach is the measurement of tissue DA content and its metabolites, 3,4-dihydroxyphenylacetic acid (DOPAC) and homovanillic acid (HVA, **Figure 1B**).

Prenatal poly I:C treatment at GD 15 induces enhanced release of DA from striatal explants in the adult offspring (83). In addition, poly I:C treatment at GD 9 results in increases DA and DOPAC levels in the PFC and the globus pallidus (GP) and HVA in the nucleus accumbens (NAcc) and GP of adult mice (133). Increases in DA are also found in the NAcc following poly I:C treatment at GD 9 (134). Similarly, several injections of poly I:C (GD 12-17) result in elevated levels of DOPAC and HVA in the adult STR (84).

Prenatal LPS treatment has been shown to have somewhat variable effects on DA. For example, daily administration of LPS throughout the entire pregnancy results in increased DA levels in the NAcc of adult animals (P 120, 170, or 400), but lower DA levels in younger animals (P 39) (119, 120). Interestingly, a single LPS administration at GD 10, results in a decrease of DA in the dSTR (135–140). Decreased DA is also found in the NAcc, PFC, amygdala, hippocampus, and hypothalamus, accompanied by decreased levels of HVA in the NAcc and amygdala (P 120) (140). Similarly, decreased DA levels in the NAcc at P 83 were found when escalating doses of LPS were administered daily from GD 15 until 19 (141).

Using MIA with TURP, we found increases in DA, DOPAC, and HVA in the NAcc, but not in the dorsal STR or the PFC of the male offspring in rats (142, 143).

Prenatal poly I:C treatment (GD 9) results in increased tyrosine hydroxylase (TH) immunoreactivity, the rate-limiting enzyme for the synthesis of DA, in the mesencephalon of embryonic mice at GD 13 and 17 (92), as well as in the NAcc and SN of adult mice (P 120) (93). In the NAcc, TH immunoreactivity was decreased at P 35 (93) but increased at P 70 (90, 93). DAT immunoreactivity is also found to be increased in the fetal mesencephalon (GD 17) (92), but decreased in the dSTR at P 35, as well as in the NAcc at GD 19 and P 35 (93). Immunoreactivity of DA receptors, D1 and D2, is reduced in the adult mice’s PFC (90, 144) and increased in the NAcc (for both D1 and D2) and dSTR (only D1) (93). In contrast, Ozawa et al. reported that DA D2 receptor’s binding is reduced in the STR of adult mice (84). Most of these data are consistent with a scenario of increased synthesis of DA in the adult brain of MIA offspring, particularly in the meso-limbic, but not in the meso-cortical pathway.

The effects of prenatal LPS administration on these markers of DA neurotransmission are rather conflicting. Borrell et al. (118) found increased TH immunoreactivity in the NAcc and bed nucleus of the stria terminalis in adult rats whose mothers were treated with LPS on alternate days during the entire pregnancy. In contrast, Ling et al. reported in several studies that a single dose of LPS at GD 10 leads to a significant decrease in TH immunoreactivity, which was significant in the SN, at several postnatal ages (P 21, 120, 210, 420, 510) as well as in the VTA of post-weanling rats (135–140). As above, these results support the idea that models of bacterial MIA have different outcomes compared to those involving viral mimetics.

Finally, prenatal TURP administration at GD 15 leads to an increase of TH levels in the NAcc, but not in other DA terminal areas such as the dorsal STR or the PFC, nor in the VTA or SN (124, 142, 143).

Overall, poly I:C and TURP induce molecular changes consistent with hyperactivity of mesolimbic DA neurotransmission, which may underlie the hyperactivity in response to AMPH and other behavioral alterations that can be corrected by administration of antipsychotics.

## Role of Maternal Cytokines in Inducing MIA Alterations

A more causal role for elevated maternal cytokines in SCZ-related alterations has been established through the administration of exogenous cytokines to pregnant rats or mice. These manipulations have been shown to be sufficient to induce several molecular and behavioral effects in the offspring. For example, prenatal administration of IL-6 in mice (at GD 9, 5 μg, i.p.) results in impairments in PPI and other behaviors in the adult offspring, whereas a similar treatment with IFNγ or TNFα does not affect the offspring (86). Significantly, the effect of an influenza virus and poly I:C treatments on the fetal brain transcriptome overlapped to those of IL-6 administration in utero, supporting the idea that many effects of poly I:C are mediated by this cytokine (145).

Importantly, functional inhibition of poly I:C-induced IL-6 in pregnant mice prevented several of the behavioral effects of prenatal poly I:C in the offspring, including impaired PPI (86). Also, the offspring of IL-6 “knock-out” mothers treated with poly I:C, do not present these alterations (86). Similarly, knock-out of IL-6 receptor in the placental trophoblasts prevented several effects of prenatal poly I:C treatment (146), indicating a crucial role of this organ in mediating the effects of MIA.

We also observed that co-treatment with an anti–IL-6 antibody during gestation and TURP prevented the development of a hyper-active DAergic system (143). This prenatal treatment effectively rescued the exaggerated AMPH-induced hyperlocomotion and behavioral sensitization, elevated DA, and TH in the NAcc in the offspring of TURP-treated mothers (143).

IL-6 can, in turn, act in more than one way to affect neurodevelopment (**Figure 1C**). One such mechanism is hypoferremia, a reduction in maternal circulating non-heme iron, which characterizes the acute phase response and is triggered by all types of infection (147, 148). Proper iron homeostasis is fundamental for healthy brain development, especially for the DAergic neurons (149). Indeed, we demonstrated that maternal iron supplementation, which counteracts inflammation-induced hypoferremia, prevented the development of exacerbated responses to a single AMPH injection and enhanced behavioral sensitization following repeated exposure to this drug in the offspring (142). Furthermore, maternal iron supplementation during MIA also reversed the increased levels of TH, DA and its metabolites in the NAcc found in the offspring of mothers treated with TURP (142). Notably, iron levels in the placenta were reduced by MIA (but not in the fetal brain), which were rescued by maternal iron supplementation (142), supporting a role for this organ in mediating the effects of MIA in the development of the brain.

Another potential mediator of MIA, downstream of IL-6, is IL-17a (**Figure 1C**), since blocking the latter cytokine with anti–IL-17a antibodies prevented cortical malformations and the emergence of abnormal behaviors in adult MIA offspring, including impaired social interaction and increases marble-burying behavior (150, 151). Meanwhile, overexpression of the anti-inflammatory cytokine IL-10 in maternal macrophages prevented the MIA-induced deficits in PPI, although in itself, elevated IL-10 also induced other behavioral alterations (91). In addition to IL-6, the hormone leptin has also been implicated on the effects of MIA in the DAergic system.

### Leptin

Leptin is the product of *ob* gene (152), a hormone that regulates food intake and energy expenditure (153–155). Leptin is primarily produced by adipose tissue and secreted into the circulation, where levels correlate positively with body fat mass (156, 157). Leptin has a multitude of physiological roles, including regulation of inflammatory processes (158, 159). For example, leptin treatment induces pro-inflammatory cytokines, including TNFα, IL-1β, IL-6, and IFN-γ (160–162). Inflammatory stimuli (e.g., TNFα, IL-1β, LPS, and TURP) in turn increase leptin synthesis (163–167). During the acute inflammatory response, leptin is involved in the induction of several sickness-type responses, such as anorexia and fever (168–173).

Despite its clear involvement in several aspects of the inflammatory response to infection, the role of leptin in brain development has not yet been extensively studied. We demonstrated that neutralization of leptin during MIA was effective in curtailing several alterations induced by prenatal TURP, including the hype-sensitized locomotor response to AMPH, and increases in DA in the NAcc (**Figure 1C**) (143). Intriguingly, leptin could affect the development of the dopaminergic system, as constitutive leptin mutant mice have impaired locomotor response to AMPH, and diminished DA release in the NAcc (174). Leptin can also exert impairing effects or the control of cytokines expression in the placenta (175).

## Concluding Remarks

MIA alters the development of the dopaminergic system and many other neurotransmitter systems and brain regions (5, 6, 79, 80). Maternal cytokines, particularly IL-6, are central in mediating these effects (5). However, other neuroendocrine factors, such as the adipokine leptin, are potentially involved and deserve further investigation.

Maternal infections and other environmental risk factors for SCZ and neurodevelopmental disorders may independently account for a few clinical cases since exposure to them does not always generate the disorder or are implicated in several psychiatric illnesses (6, 39, 79, 80, 176, 177). In this regard, heterogeneity of response characterizes all known environmental risk factors for psychopathology, including the most overwhelming of traumas (176). Such response heterogeneity is associated with pre-existing genetic (175) or epigenetic (i.e., chromatin modifications) differences (178).

This hypothesis implies that in any given population, individual predisposition is directly responsible for the vulnerability or resilience to the environmental causes of many psychiatric conditions (176), including SCZ (39, 179). Regarding vulnerability, there is a significant interaction between maternal HSV‐2 seropositivity and *GRIN2B* genetic variation (*GRIN2B* encodes for a NMDA glutamate receptor) (180). Also, exposure to maternal infection has been reported to increase the risk of SCZ only in cases with a family history of psychiatric disorders (181, 182). Animal models of MIA support this notion, as the effects of poly I:C are enhanced when they occur in mice mutant for genes linked to SCZ and other disorders (183–185). Furthermore, interaction with other environmental risk factors, such as maternal diet, gut microbiota, or experiences of peripubertal trauma, can have a synergistic effect with maternal infection or prevent its detrimental effect (6, 79, 186). Therefore, systematically generating translational models of the interaction between genetic and environmental (or environmental and environmental) risk factors for SCZ and other neurodevelopmental and psychiatric disorders appears to be the next step in understanding the etiology of mental illnesses.

## Author Contributions

AA-V, BR, and EM-C wrote and edited the manuscript.

## Conflict of Interest

The authors declare that the research was conducted in the absence of any commercial or financial relationships that could be construed as a potential conflict of interest.
